# Spectral-aware CNN with learnable biorthogonal units and depthwise convolutions for multi-class blood cell classification

**DOI:** 10.1016/j.mex.2025.103685

**Published:** 2025-10-24

**Authors:** Sannasi Chakravarthy SR, Harikumar Rajaguru, Rajesh Kumar Dhanaraj, Feslin Anish Mon, Dragan Pamucar

**Affiliations:** a Department of Electronics and Communicaiton Engineering, Bannari Amman Institute of Technology, Sathyamangalam 638 401, India; b Symbiosis Institute of Computer Studies and Research (SICSR), Symbiosis International (Deemed University), Pune, India; c Faculty in department of Information technology, University of Technology and Applied Sciences, Ibri, Oman; d Széchenyi István University, Győr, Hungary

**Keywords:** Blood cell classification, Deep learning, Wavelet CNN, Spectral-aware downsampling, Depthwise separable convolution

## Abstract

For effective and early diagnosis of diseases such as leukemia and anemia, accurate classification and interpretation of peripheral blood cells are critical. A novel hybrid deep learning model is proposed in this study for multi-class blood cell classification, called Spectral-Aware CNN with Learnable Spectral Biorthogonal Downsampling Units (LSBDUs) and Depthwise Separable Convolutions. The model replaces conventional pooling layers with wavelet-inspired LSBDUs for improved feature retention. This results in reduced computational overhead through efficient separable convolutions. The research used a balanced dataset of 17,092 images across eight blood cell classes. The techniques, such as stratified data splitting, advanced augmentation, and label smoothing, are included in the training pipeline for improving generalizability. As a result, the model achieves 99.18 % of overall classification accuracy with superior class-wise performance.

• Replaces pooling layers with spectral-aware LSBDU blocks for better feature preservation.
• Integrates Depthwise Separable Convolutions to reduce parameter count and training cost.
• Demonstrates superior generalization across all classes without overfitting.

## Graphical abstract

**Figure:**
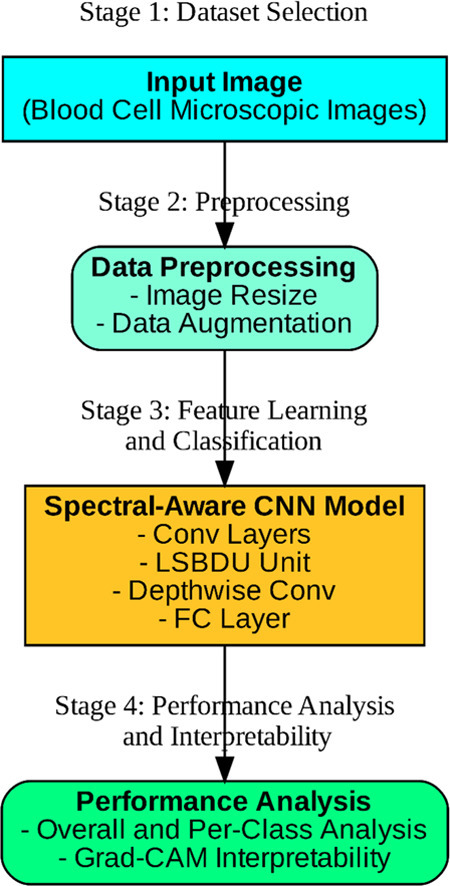

**Specifications table**

**Table:** 

**Subject area**	Engineering
**More specific subject area**	*The study focuses on improving the performance of Convolutional Neural Networks for blood cell classification using wavelet and depthwise separable convolutions.*
**Name of your method**	*Spectral-Aware CNN with Learnable Spectral Biorthogonal Downsampling Units (LSBDUs) and Depthwise Separable Convolutions*
**Name and reference of original method**	*None*
**Resource availability**	*Blood Cells Image Dataset* https://www.kaggle.com/datasets/unclesamulus/blood-cells-image-dataset *Raabin-WBC Data:*https://raabindata.com/

## Background

The problem of peripheral blood cell classification is a fundamental task in hematological diagnostics. This plays a vital role in the identification of infections, immune disorders, and various forms of leukemia [1]. In practice, this process is traditionally conducted in a manual manner. This utilizes the help of trained pathologists who examine blood smear slides under a microscope [2]. Although this approach is found to be effective, it is a time-consuming process [3]. Also, it is prone to inter-observer variability and thus limited by the availability of expert personnel [4]. This will become a challenge, particularly in rural healthcare or under-resourced settings. This leads to a growing demand for automated, computer-aided solutions that deliver faster, more accurate, and more consistent results. Recently, deep learning (DL) techniques have shown significant promise in medical image analysis. The DL techniques outperform conventional machine learning (ML) approaches in many visual recognition tasks. However, a challenge remains in balancing model complexity with performance. Many state-of-the-art convolutional neural networks (CNNs), such as DenseNet, achieve higher accuracy but at the cost of high computational requirements [5]. This can make them impractical for deployment in real-time or resource-limited environments, such as portable diagnostic tools or point-of-care systems [6].

The study is intended to design a highly accurate deep learning model that is tailored specifically for the multi-class blood cell classification task. The model proposed in the study retains important spatial and spectral information with the advantage of reducing unnecessary computational overhead. In this way, the study presents a novel approach combining two powerful ideas. The first one involves spectral-aware downsampling through Learnable Spectral Biorthogonal Downsampling Units, and then efficient feature extraction is done using Depthwise Separable Convolutions. This involves the replacement of conventional pooling operations with LSBDUs. This enables the model to preserve more nuanced features in the spectral domain, which are often lost in standard max or average pooling. In particular, this is significant for fine-grained classification tasks, such as the employed blood cell classification, where differences between classes may be subtle. In the meantime, Depthwise Separable Convolutions [7] are employed for reducing the number of trainable parameters without compromising representational power. Thus, making the proposed model to remain efficient and suitable for effective clinical settings. In addition to the above discussion, another key motivation is to ensure the model performs well across all classes, not just the dominant majority classes. This problem is addressed using techniques such as stratified data splitting [8], advanced augmentation, and focal loss [9]. These techniques facilitate in such a way that all of them contribute to better generalization and robust performance.

In addition to the above discussion, several prior works have explored the combination of frequency-domain ideas with convolutional networks [15,16]. This includes approaches that use Wavelet transforms as pre-processing or apply fixed wavelet filters inside networks. Herein, the works that employ wavelet filtering typically rely on hand-crafted or fixed wavelet bases (applied as a pre-processing step or as non-trainable layers). These works employed these techniques to supply frequency-domain features to a subsequent CNN model. Other spectral approaches (for example, Fourier-domain or spectral pooling variants) commonly operate on global frequency coefficients. Moreover, they often require explicit truncation or global spectral manipulation, which can sacrifice spatial locality and interpretability. Thus, the proposed Learnable Spectral Biorthogonal Downsampling Unit (LSBDU) differs from these prior directions in the following three important ways:

(i) LSBDU implements learnable biorthogonal low-pass and high-pass filters that are trained jointly with the network weights. This is quite rather than fixed wavelet bases applied externally. Unlike fixed Wavelet pre-processing, this enables the model to adapt spectral filters specifically to the employed problem.
(ii) LSBDU is integrated into the network as a replacement for pooling (applied per feature-channel with strided convolutions). This preserves both spatial locality and spectral decomposition. This differs from methods that perform global spectral pooling or operate on whole-image Fourier coefficients.
(iii) LSBDU learns scalar fusion weights (α,β) that adaptively combine low- and high-frequency components per channel. This enables the network to emphasize the spectral modes most helpful to downstream classification. Notably, the LSBDU produces spectral attention maps that improve interpretability as discussed in the sub-section “*Interpretability of the Proposed Model*”.

The aforementioned distinctions reveal why LSBDU provides superior task-specific spectral feature extraction while remaining compatible with efficient spatial operators such as depthwise separable convolutions. Furthermore, Table 1 illustrates the qualitative comparison between prior wavelet-CNN/spectral strategies and the work’s Learnable Spectral Biorthogonal Downsampling Unit (LSBDU) methodology. In this way, the proposed methodology aims to bridge the gap between clinical need and technological capability. Thus, the study presents a novel methodology that is not only accurate but also efficient and interpretable.

**Table 1:** Comparison of spectral/frequency methodologies.

Methodology	Spectral Filters	Integration Point in Network	Per-channel learnable	Spatial Locality	Interpretability
Fixed Wavelet + CNN hybrids [34,35]	Fixed and hand-crafted	Pre-processing or non-trainable wavelet layers	No	Preserved	Moderate
Global spectral / Fourier methods [13,40]	Global frequency coefficients	Transform layer or pooling in the frequency domain	Rarely	Reduced	Low-moderate
Spectral pooling variants [41,42]	Truncated spectrum or spectral attention masks	Spectral pooling or attention module	No	Reduced	Low
Proposed LSBDU	Learnable biorthogonal filters (per channel)	Integrated downsampling layer (replaces pooling)	Yes	Preserved	High (spectral attention maps)

## Method details

This section examines the employed database and provides a detailed discussion on the proposed architecture of the study.

### Dataset used

**Fig. 1:**
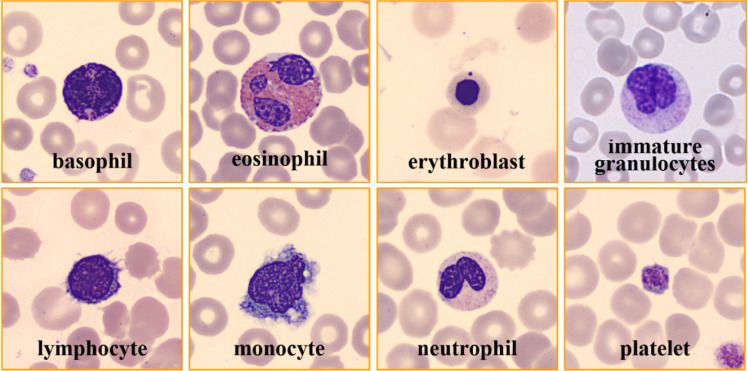
Sample blood cell images from the kaggle dataset (8 Classes).

**Fig. 2:**
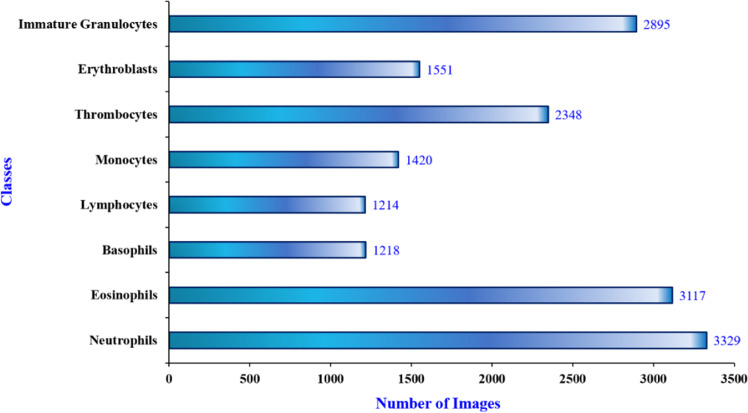
Dataset distribution for 8 classes.

The study begins with a data collection phase, during which the publicly available Blood Cell Image Dataset [10] from Kaggle is used. The dataset contains 17092 images of individual normal cells. These images were acquired at the Hospital Clinic of Barcelona’s Core Lab. The data acquisition involves the use of the CellaVision_DM96 analyzer. It consists of images separated into 8 distinct classes: thrombocytes (platelets), neutrophils, erythroblasts, eosinophils, monocytes, lymphocytes, basophils, and immature granulocytes (metamyelocytes, myelocytes, and promyelocytes). The images in the dataset are of resolution 360×363 pixels. And all these images are available publicly in JPEG format. In addition, the annotation of images in the dataset was carefully done by experienced clinical pathologists. Here, all the scans were acquired from healthy people with no infections, blood diseases, or cancer, and they were not taking any medication when their blood was collected. The sample images from each class of the blood cell image dataset are illustrated in Fig. 1. The dataset distribution of eight classes is illustrated in Fig. 2. As from this Figure, the study poses a challenge of class-imbalance since the classes such as Basophils, Lymphocytes, Monocytes, and Erythroblasts have considerably fewer samples as compared with the Neutrophils and Eosinophils classes. These characteristics of skewed distributions can make the model to learn biased. In addition to this, as from Fig. 1, there exists a high degree of intra-class variability and inter-class visual similarity. Thus, these challenging factors in the study necessitate a more discriminative model architecture capable of capturing fine-grained differences in spatial and spectral patterns.

### Data pre-processing and augmentation

**Fig. 3:**
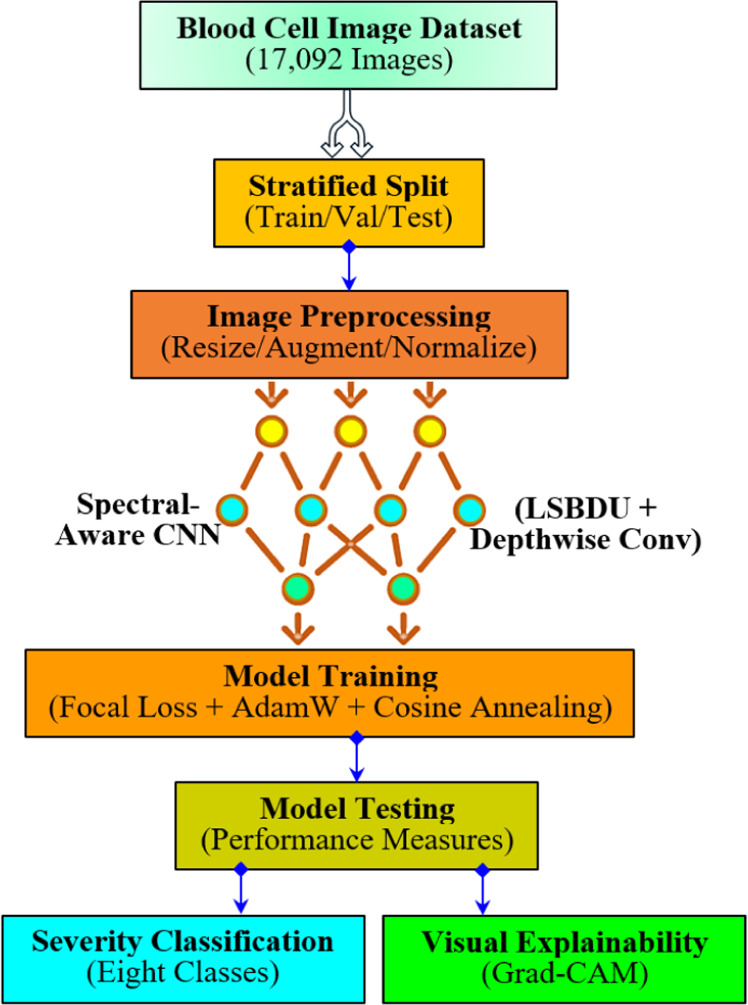
Overall workflow.

Fig. 3 illustrates the overall workflow of the proposed framework. As shown in the Figure, all blood cell images in the dataset are processed through a consistent pre-processing pipeline to standardize input dimensions and enhance dataset diversity. At first, all images are resized to 224×224 pixels in order to match the input resolution of the convolutional neural network models. Then, a suite of data augmentation techniques is applied to expand the dataset artificially. This leads to enhance the model's robustness to real-world variability. This way of augmentation [11] involves random horizontal and vertical flips, random rotations, color jittering (adjustments to brightness, contrast, saturation, and hue), and random affine transformations to simulate variations in cell orientation and microscope angle. In addition to this, the study employs cutout augmentation to randomly mask rectangular regions of images. This facilitates the model to learn discriminative features beyond localized regions. Next, the images are normalized using the mean and standard deviation values that are computed across the dataset channels. This ensures consistency in input data distribution. Also, normalization [12] helps accelerate convergence and improve numerical stability. Notably, the study augments only the training set, whereas the validation and test sets are strictly normalized with no random transformations. This enables the study to maintain a fair evaluation procedure. Thus, these pre-processing strategies, as shown in Fig. 3, involving stratified data splitting, contribute to better generalization and reduced overfitting. In particular, this is helpful in the presence of class imbalance and higher intra-class visual similarity.

### Proposed CNN architecture

**Fig. 4:**
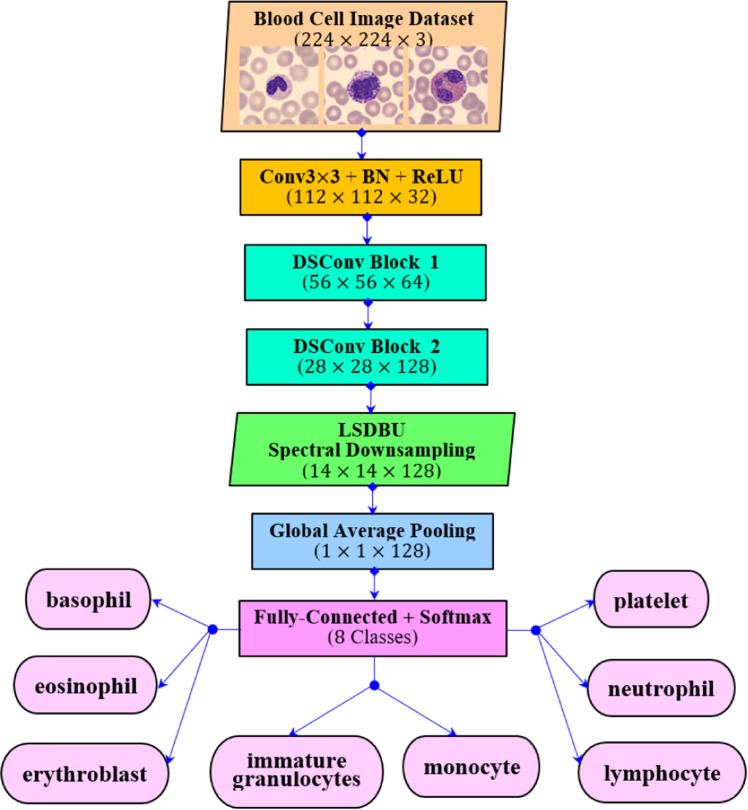
The proposed spectral-aware CNN with depthwise connections and LSDBU units.

**Fig. 5:**
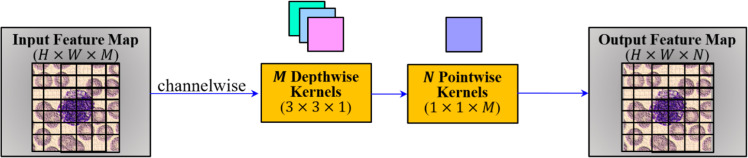
Depthwise separable convolution (DSC).

As illustrated in Fig. 1, Fig. 2, the study poses the challenges of class imbalance and subtle inter-class visual similarity in blood cell images. In order to tackle this, the study proposes a Spectral-Aware Convolutional Neural Network (CNN) model for blood cell image classification. The proposed CNN architecture for the employed problem is illustrated in Fig. 4. Here, the architecture integrates two core innovations, namely Depthwise Separable Convolutions and the Learnable Spectral Biorthogonal Downsampling Unit (LSBDU). As shown in Fig. 4, the model involves the combination of spectral decomposition into the learning pipeline while maintaining computational efficiency through the use of depthwise separable convolutions, which is inspired by MobileNet. The architecture consists of an initial convolutional block performing a 3×3 convolution, batch normalization, and a ReLU activation. Then the network is followed by a series of Depthwise Separable Convolution (DSC) layers. The illustration of depthwise separable convolution used in the study is given in Fig. 5. As shown, these DSC blocks help the architecture to separate the spatial and channel-wise filtering operations. This results in a significant reduction in computational load as compared with the standard convolutions [13]. The mathematical representation of the computational cost of a standard convolution is given in Eq. (1) [13].

(1) $$Cost_{Standard}=D_{K}^{2}.M.N.D_{F}^{2}$$

In Eq. (1), $D_{K}$, $D_{F}$, M, and N represent the kernel size, spatial size of the feature map, number of input channels, and output channels. For the same, the mathematical representation of depthwise separable convolutions is given in Eq. (2) [14].

(2) $$Cost_{DSC}=D_{K}^{2}.M.D_{F}^{2}\;+\;M.N.D_{F}^{2}$$

The optimization, as mentioned in Eq. (2), enables the model to handle high-resolution inputs with fewer parameters, resulting in faster, more effective training.

**Fig. 6:**
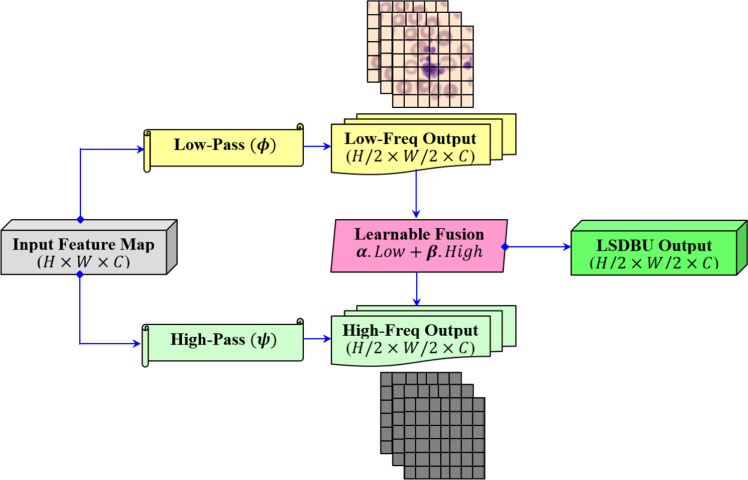
Illustration of the learnable spectral biorthogonal downsampling unit (LSBDU) used in the spectral-aware CNN.

The proposed method’s distinguishing feature is the Learnable Spectral Biorthogonal Downsampling Unit (LSBDU). This unit simply replaces the traditional max or average pooling as used in the standard CNNs. Unlike fixed pooling mechanisms, this LSBDU employs learnable low-pass and high-pass biorthogonal filters [15]. This is adopted in the study for decomposing input features into spectral components. The illustration of the LSBDU used in our study is given in Fig. 6.

As shown in Fig. 6, the LSDBU unit performs spectral filtering that is introduced directly within the CNN architecture, as illustrated in Fig. 4. For an input feature map, $X\in R^{H\times W\times C}$, the LSDBU unit applies learnable biorthogonal low-pass (ϕ) and high-pass (ψ) filters per channel [16]. This is mathematically represented as given in Eqs. (3) and (4).

(3) $$L_{c}=X_{c}*\phi_{c}$$

(4) $$H_{c}=X_{c}*\psi_{c}$$

In Eqs. (3) and (4), the symbol * denotes the operation of convolution, where $L_{c}$ and $H_{c}$ are the resultant low and high-frequency components for channel c. Here, each spectral component is downsampled by 2 as shown in Fig. 6. This downsampling is performed by a factor of 2 using strided convolution. This can be mathematically represented in Eq. (5).

(5) $${\overset{⌣}{L}}_{c}=Downsample\left(L_{c}\right),\;{\overset{⌣}{H}}_{c}=Downsample\left(H_{c}\right)$$

After spatial downsampling of these components, adaptive fusion using learnable scalar weights (α,β) is performed, which enables the model to emphasize either spectral mode based on the downstream task. This can be mathematically represented as given in Eq. (6).

(6) $$Y_{c}=\alpha_{c}.{\overset{⌣}{L}}_{c}+\beta_{c}.{\overset{⌣}{H}}_{c}$$

During the implementation, $\phi_{c}$ and $\psi_{c}$ are per-channel 2D kernels of size 3×3 applied through grouped convolutions (groups = C). The Downsample operator is implemented as a strided convolution with stride s=2 and same padding so that ${\overset{⌣}{L}}_{c},{\overset{⌣}{H}}_{c}\in R^{H/2\;\times W/2}$. Here, the fusion coefficients are learned per-channel and constrained to (0,1) using a sigmoid as given in Eq. (7).

(7) $$\alpha_{c}=\sigma \left(a_{c}\right),\;\;\beta_{c}=\sigma \left(b_{c}\right)$$

In Eq. (7), $a_{c}$ and $b_{c}$ are the trainable scalars. Here, the filters $\phi_{c}$ and $\psi_{c}$ are initialized with biorthogonal wavelet coefficients (Bior2.2) and updated during training. All convolutional padding, kernel sizes, and initialization details are reported in Table 2. Finally, these components are stacked across the channels for obtaining the output as represented in Eq. (8).

(8) $$Y=Concat\left(Y_{1},Y_{2},\ldots ,Y_{C}\right)$$

**Table 2:** Hyperparameters for training the proposed spectral-aware CNN Model.

Hyperparameters	Values
Input image size	224×224×3
Batch size	32
Epochs	50 (with Early Stopping enabled at patience = 5)
Optimizer	AdamW
Initial learning rate	0.001
Learning rate scheduler	Cosine Annealing with warm restarts
Weight decay	1e-4
Loss function	Focal Loss with α=0.25, γ=2
Label smoothing	0.1 (applied in target label encoding)
Downsampling Strategy	Learnable Spectral Biorthogonal Downsampling Unit (LSBDU)
Evaluation Metrics	Confusion Matrix, Accuracy, Precision, Recall, F1-score, and ROC-AUC

The above process of Equations from (3) to (6) enables the network to adaptively emphasize spectral features that are important for the employed classification task. Finally, the feature maps are then passed through a Global Average Pooling (GAP) [17] layer. This layer is introduced here for reducing spatial dimensions. Next, a fully connected layer [18] and a Softmax [19] activation function are introduced into the architecture to produce class probabilities, as given by Eq. (9).

(9) $${\hat{y}}_{i}=\frac{\text{exp}\left(z_{i}\right)}{\sum_{j=1}^{C}\text{exp}\left(z_{j}\right)}\;for\;i=1,\ldots ,C$$

In Eq. (9), C represents the number of output classes, which is 8 for the employed classification problem. And $z_{i}$ denotes the logit for class i. Thus, the complete pseudocode for the forward propagation through LSDBU and the classification head is illustrated in Algorithm 1 below.

**Algorithm 1:** Forward propagation through LSBDU and classification head.

Input:
$X\;\in \;R^{\left\{H\times W\times C\right\}}$
Parameters:
{$\phi_{c}$, $\psi_{c}$} for c=1..C
$\;\{a_{c},\;b_{c}\}\;for\;c=1..C$
Downsample()
DSC_block()
FC, Softmax()
Output:
$\hat{y}\;\in \;R^{\left\{C\right\}}$
Procedure:
1: for c=1toC do
2: *#Spectral filtering (*Eqs 3*-*4*):*
3: $L_{c}\;\leftarrow$ Conv2D(input = $X_{c}$, kernel = $\phi_{c}$, groups=1, padding='*same*') representing low-pass
4: $H_{c}\leftarrow$ Conv2D(input = $X_{c}$, kernel = $\psi_{c}$, groups=1, padding='*same*') representing high-pass
5: *#Spatial downsampling* (Eq. 5):
6: ${\overset{⌣}{L}}_{c}\;\leftarrow$ Downsample($L_{c}$)
7: ${\overset{⌣}{H}}_{c}\leftarrow$ Downsample($H_{c}$)
8: *#Adaptive fusion (*Eq. 6*):*
9: $\alpha_{c}\;\leftarrow$ sigmoid($a_{c}$) with constrains $\alpha_{c}\in \left(0,1\right)$
10: $\beta_{c}\;\leftarrow$ sigmoid($b_{c}$) with constrains $\beta_{c}\in \left(0,1\right)$
11: $Y_{C}\leftarrow \;\alpha_{c}.{\overset{⌣}{L}}_{c}+\beta_{c}.{\overset{⌣}{H}}_{c}$
12: end for
13: *#Reconstruct channel tensor (*Eq. 7*):*
14: Y ← Concat_channels($Y_{1}$, $\;Y_{2}$, …, $\;Y_{C}$) with $Y\in R^{H/2\;\times W/2\;\times C}$
15: Y' ← DSC_block(Y) for Depthwise separable conv refinement
17: Z ← GAP(Y') with Global Average Pooling → shape [C]
18: z ← FC(Z) representing logits per class
19: $\hat{y}\leftarrow \;Softmax\left(z\right)$ as shown in Eq. (9)
20: return $\hat{y}$

As a final point of summary, the proposed Spectral-Aware CNN model is tailored for achieving robust classification performance. This has been achieved by unifying frequency-domain learning with LSBDU and by efficiently extracting spatial-channel features via depthwise separable convolutions. This combination helps the proposed model to provide both accuracy and interpretability. Thus, making the proposed architecture well-suited for the employed fine-grained blood cell classification task.

## Method validation

This section presents the discussion on implementation, experimentation, and results of the proposed study.

### Experimental setup and training of the proposed study

The hardware specifications for the implementation of the proposed model are as follows: the IDE used is Python 3.6, which runs on a desktop computer having 32 GB RAM, an Intel Core i7 processor, a 1 TB hard disk, and an NVIDIA RTX 3090 GPU. The hyperparameter specifications used for training the proposed spectral-aware CNN are summarized in Table 2.

**Fig. 7:**
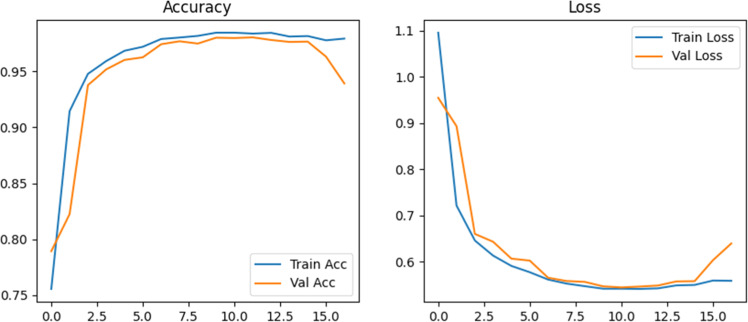
Learning curves of the proposed model.

As summarized in Table 2, the standard evaluation metrics [20] are employed for performing a comparative investigation on the performance of the proposed model. In addition to these metrics, the study employs Cohen’s kappa score [21] for further validating the model. The proposed Spectral-Aware CNN’s training and validation behavior is analyzed over 17 epochs. Here, the experimentation is conducted with an early stopping strategy [22] in order to prevent overfitting. The training process is optimized using the AdamW optimizer [23]. This combines with focal loss to address class imbalance and label smoothing [24] to regularize the classifier. And the learning rate is scheduled using Cosine Annealing [25]. This helps in promoting better convergence in later epochs of the training phase. The obtained training and validation plots are illustrated in Fig. 7.

The left part of Fig. 7 illustrates the learning curves of training and validation accuracy, which indicates a smoother and stable convergence. As shown in the Figure, the training accuracy steadily improved from 75.5 % in epoch 1 to 99.6 % by epoch 10. This is also increasing with a corresponding rise in validation accuracy from 78.9 % to 99.0 %. Here, it is noted substantially that the validation performance of the model has not diverged significantly from training. This confirms that the proposed model generalizes well without overfitting. Now, the right part of Fig. 7 illustrates the evolution of training and validation losses of the proposed model. In the plot, it is noted that the loss consistently decreased with no signs of oscillations or any rapid improvement. This signifies the stability of the proposed model under the chosen training configuration. Herein, the early stopping mechanism is enabled at epoch 17. This mechanism is based on validation loss stagnation and thus ensures training efficiency. The above-discussed observations confirm that the proposed framework, including effective augmentation, wavelet-aware downsampling (LSBDU), and a robust training pipeline, achieves efficient convergence and excellent generalization to unseen blood cell images.

### Classification performance analysis

**Fig. 8:**
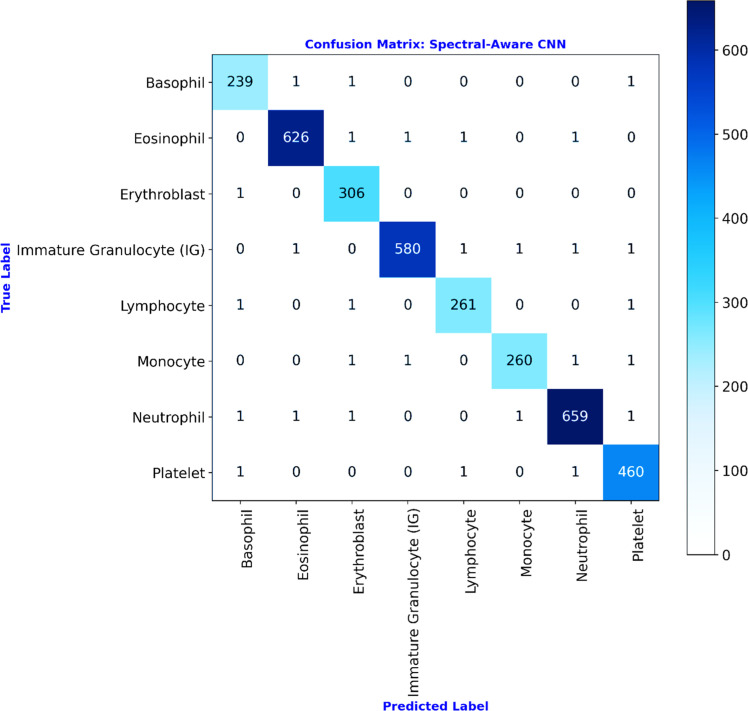
Confusion matrix for the testing phase of the proposed model.

In order to ensure fair evaluation across all eight classes of the input dataset, the data is split using stratified sampling, where 80 % for training/validation and 20 % for the testing set. This way of stratified data splitting preserves class distributions. That is, this way of data partition ensures that at least 240 samples per class should be present in the test set, which is sufficient for stable multi-class evaluation metrics. After training, the confusion matrix obtained for the testing phase is illustrated in Fig. 8. Based on this matrix, the overall performance of the proposed model is listed in Table 3.

**Table 3:** Overall classification performance comparison of the proposed model.

Models	Overall Accuracy ( %)	Misclassification Rate	Macro-F1 Score ( %)	Cohen’s Kappa Validation Score (0 to 1)
Custom CNN with MaxPooling	95.42	0.0458	94.76	0.950
VGG16	96.15	0.0385	95.88	0.960
ResNet18	96.92	0.0308	96.75	0.968
MobileNetV2	97.33	0.0267	97.22	0.970
EfficientNet-B0	97.48	0.0252	97.05	0.972
ConvNeXt-Tiny	97.95	0.0205	97.62	0.981
Vision Transformer (ViT-B/16)	98.24	0.0176	97.89	0.985
Proposed Spectral-Aware CNN with LSBDUs	99.18	0.0082	99.08	0.99

**Fig. 9:**
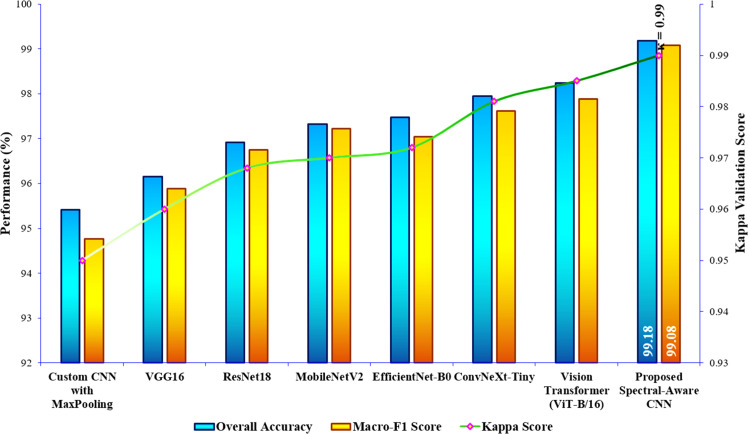
Graphical performance comparison of the proposed model.

For assessing the proposed Spectral-Aware CNN’s efficiency, the study compared its performance against several established deep learning architectures. This includes a custom CNN with max-pooling operation [26], VGG16 [27], ResNet18 [28], MobileNetV2 [29], EfficientNet-B0 [46], ConvNeXt-Tiny [47], and Vision Transformer (ViT-B/16) [48]. Herein, all baseline models are trained under the same experimental conditions as the proposed Spectral-Aware CNN to ensure a fair and reproducible comparison. It is clarified that no model received any dataset-specific tuning or additional augmentation beyond these common settings (Table 2). All random seeds, data splits, and training logs are fixed and archived for reproducibility. This way of performance assessment ensures that the observed performance differences in Table 3 directly reflect the architectural merits of each model rather than discrepancies in hyperparameter optimization or preprocessing strategies. The graphical comparison of the overall performance analysis of the proposed model is illustrated in Fig. 9. As given in Table 3 and Fig. 9, the Spectral-Aware CNN with LSBDUs attained the highest overall accuracy of 99.18 %, significantly outperforming other deep learning models. Along with this overall accuracy, the proposed model provides a macro-F1 score of 99.08 %. This indicates the attainment of balanced precision and recall across all classes, irrespective of the presence of mild class imbalance in the employed dataset. In addition, it is noted that the misclassification rate is as low as just 0.82 %. This further confirms the robustness of the proposed model's predictions. In comparison to the proposed model’s performance, traditional models such as VGG16 and ResNet18 achieved lower accuracies of 96.15 % and 96.92 %, respectively, with correspondingly higher misclassification rates and lower F1-scores. In addition, the recent models, EfficientNet-B0, ConvNeXt-Tiny, and ViT-B/16, provided a better range of classification accuracy from 97.48 % to 98.24 %. Specifically, the MobileNetV2 architecture employing depthwise separable convolutions as similar to the proposed model, performed relatively well (97.33 % accuracy). But the architecture still fell short of the Spectral-Aware CNN’s performance. This, in turn, emphasizes the added value of incorporating Learnable Spectral Biorthogonal Downsampling Units (LSBDU) in the proposed architecture. Additionally, a robust statistical measure of inter-rater agreement, called the Cohen’s Kappa score, is used for further validating the model’s reliability. As a result, the Spectral-Aware CNN with LSBDU achieved 0.99 as a kappa score. This validation score signifies near-perfect agreement between predicted and ground truth labels for the employed classification task. As a final point of summary, the substantial improvement across all performance metrics highlights the superiority of integrating spectral-aware features and optimized downsampling strategies in medical image classification tasks.

**Fig. 10:**
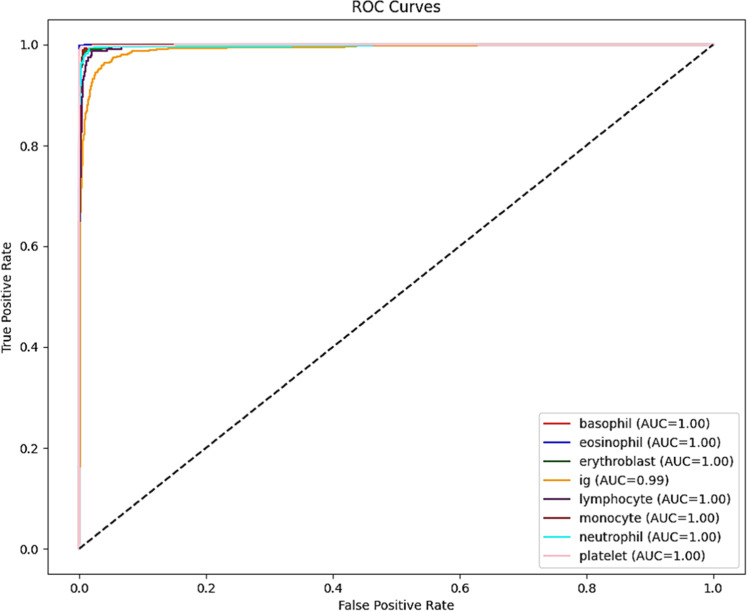
Receiver operating characteristic (ROC) curves for each blood cell class using the proposed spectral-aware CNN.

The class-wise evaluation metrics of the proposed Spectral-Aware CNN are given in Table 4. This highlights that the proposed model consistently provides higher performance across all eight blood cell severities. It is noted that precision, recall, and F1-scores remain above 98 % for all classes. This indicates the ability of the proposed model to accurately distinguish between morphologically similar cell types. In particular, the classes such as Eosinophils, Neutrophils, and Immature Granulocytes attained F1-scores exceeding 99.3 %, demonstrating exceptional classification performance. In addition, the specificity values are obtained above 99.8 % for all classes. This confirms the strong capability of the proposed model to correctly identify negative cases without any false alarms. The ROC curves in Fig. 10 illustrate the proposed model’s class-wise discriminatory power. The proposed Spectral-Aware CNN achieved AUC = 1.00 for seven out of eight classes. And an AUC of 0.99 for the Immature Granulocytes class, which indicates near-perfect classification capability. This strongly reinforces the model's ability to distinguish between visually similar cell types with minimal false positives.

**Table 4:** Class-wise performance analysis of the proposed model.

Class	Precision ( %)	Recall ( %)	F1-Score ( %)	Specificity ( %)
Basophil	98.35	98.76	98.56	99.87
Eosinophil	99.52	99.37	99.44	99.89
Erythroblast	98.39	99.67	99.03	99.84
Immature Granulocyte	99.66	99.15	99.4	99.93
Lymphocyte	98.86	98.86	98.86	99.9
Monocyte	99.24	98.48	98.86	99.94
Neutrophil	99.4	99.25	99.32	99.85
Platelet	98.92	99.35	99.14	99.83

### Interpretability of the proposed model

**Fig. 11:**
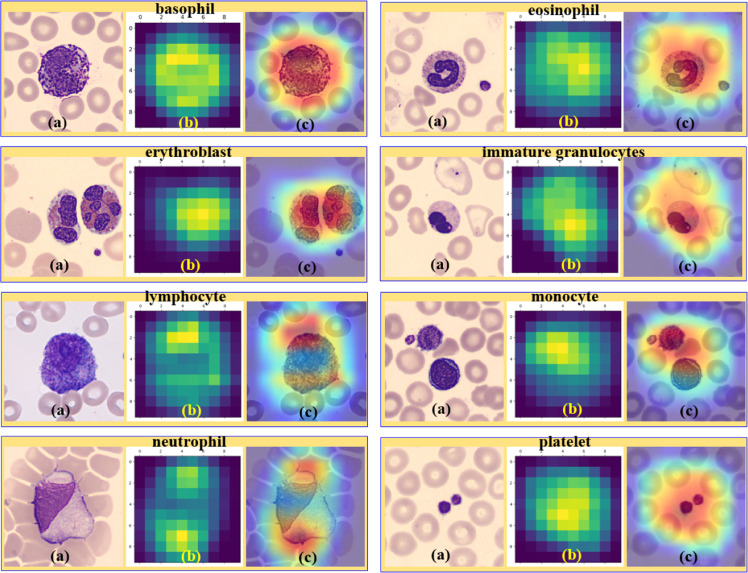
Grad-CAM visualizations for each of the eight blood cell classes. Each row depicts (a) the original input image, (b) the corresponding LSBDU-learned spectral attention heatmap, and (c) the Grad-CAM overlay highlighting the most discriminative regions used by the proposed model for classification.

Fig. 11 illustrates the sample Grad-CAM visualizations [30,31], which validate the interpretability of the proposed Spectral-Aware CNN model. As shown here, the model consistently focuses on morphologically relevant regions for each class. In particular, the visualization reveals that the model focuses on nuclear textures and cytoplasmic patterns while ignoring irrelevant background information. In Fig. 11, the LSBDU-derived spectral attention map is presented in the center panel (b) of each grid. This (b) part reveals that the biorthogonal filtering helps in preserving salient features during downsampling. And the final heatmap (c) of the image grid represents the effective overlay of the class activation map onto the original input. This, in turn, confirms that the proposed model not only achieves high classification accuracy but also provides biologically meaningful visual explanations. As a final point of view, the visualizations given in Fig. 11 reinforce the robustness and transparency of the proposed architecture.

### External dataset validation

An external validation experiment is conducted in the research to further evaluate the robustness and cross-domain generalization of the proposed Spectral-Aware CNN model. External validation is performed using two datasets: the Raabin-WBC [43] and the Barcelona [44] datasets. The first dataset, Raabin-WBC, comprises high-resolution white blood cell (WBC) images obtained from multiple laboratories under varied staining conditions and microscope setups. The second dataset, Barcelona, includes individual normal cell images acquired at Barcelona Clinic using the CellaVision DM96 analyzer. The first dataset contains five classes: neutrophils, eosinophils, monocytes, lymphocytes, and basophils. The second dataset has eight classes similar to the primary one. All images from both datasets were resized to 224 × 224 × 3 and normalized using the same mean and standard deviation statistics as the primary training dataset.

To ensure a fair comparison, no additional augmentations are employed, but stain normalization using the Macenko method [45] is performed to mitigate inter-laboratory color variations. In the Raabin-WBC dataset, 200 blood cell images from each of the five classes, and in the Barcelona dataset, 1200 blood cell images from each of the eight classes have been employed. In this way, the external validation is performed through two protocols, namely zero-shot training (the proposed model trained already on the primary dataset without any retraining or adaptation) and fine-tuning (the pretrained model is further optimized using a small fraction (15 % of samples per class) from the respective external dataset, while testing was carried out on the remaining unseen data). Herein, the first protocol setting measures the ability of the proposed Spectral-Aware CNN to generalize to unseen data domains. And the second protocol setting evaluates how quickly the model adapts to new clinical imaging conditions.

The results summarized in Table 5 illustrate that the proposed Spectral-Aware CNN demonstrates strong robustness and adaptability across both external datasets. In the first evaluation strategy, the obtained accuracies of 94.82 % on the Raabin-WBC dataset and 95.43 % on the Barcelona dataset indicate that the learnable spectral units preserve discriminative spectral-spatial information even under different staining and imaging conditions. In the next evaluation strategy, after fine-tuning with only 15 % of external samples per class, the accuracies improved to 97.56 % and 98.12 %, respectively. As a final point of conclusion, the results in Table 5 confirm that the proposed architecture maintains high generalization capability while requiring minimal adaptation effort.

**Table 5:** External dataset validation results of the proposed spectral-aware CNN.

External Datasets	Evaluation Strategy	Overall Accuracy ( %)	Macro-F1 Score ( %)	Cohen’s Kappa Validation Score (0 to 1)
Raabin-WBC Dataset (5 classes: Neutrophil, Eosinophil, Monocyte, Lymphocyte, Basophil)	Zero-Shot Testing	94.82	93.97	0.94
	Fine-Tuned (15 % data per class)	97.56	97.01	0.97
Barcelona Dataset (8 classes identical to the primary dataset)	Zero-Shot Testing	95.43	94.82	0.95
	Fine-Tuned (15 % data per class)	98.12	97.84	0.98

### Comparison with state-of-the-art methods

As a final point for comparing the proposed model with other existing works similar to the multi-class classification of blood cells, the study summarizes the recent works in Table 6. As from the Table, the proposed work outplays the recent models on the employed eight-class classification task. Table 7 summarizes the comparative analysis of the total number of learnable parameters and FLOPS. From this table, it is evident that the proposed model is only slightly more complex than MobileNetV2, which uses simple depthwise separable convolutions without any spectral learning component. However, the proposed Spectral-Aware CNN incorporates Learnable Spectral Biorthogonal Downsampling Units (LSBDUs) that enhance feature discrimination with only a modest increase in computational cost. Thus, the proposed model effectively balances spectral feature richness and computational efficiency, demonstrating an excellent trade-off between accuracy and complexity, as shown in Tables 3 to 7.

**Table 6:** Comparison of the proposed model with existing models.

Work	Year	Architecture	Overall Accuracy ( %)
[32]	2019	Transfer learning models	90
[33]	2019	CNN with object detection models	93.1
[34]	2020	CNN with Deep Convolutional Generative Adversarial Networks	91.7
[35]	2022	CNN with machine learning models	88.8
[36]	2023	CNN model	90.1
[37]	2023	R-CNN with transfer learning	95
[38]	2024	Transfer learning approach	94.7
[39]	2024	Hybrid neighborhood component analysis model	95.6
Proposed Model		Spectral-Aware CNN with Learnable Biorthogonal Units and Depthwise Convolutions	99.18

**Table 7:** Computational complexity analysis.

Model	Parameters (M)	GFLOPs	Train time/epoch (s)
Custom CNN (Max Pooling)	2.10	2.50	208
VGG16	138	16	1290
ResNet18	12	2	150
MobileNetV2	3.47	0.30	25
EfficientNetB0	5.28	0.39	33
ConvNeXt-Tiny	28.60	4.50	375
ViT-B/16	86.60	17.60	1466
Proposed Spectral Aware CNN	4.26	1.57	131

## Limitations

• Dataset Size and Diversity: Although the dataset used is a balanced and sufficient one for the current task, it might not represent all population variability conditions, such as rare morphological abnormalities or imaging conditions found in real-world clinical settings.
• Computational Complexity during Training: Although depthwise separable convolutions reduce inference-time complexity, the LSBDU module’s inclusion causes additional training-time overhead due to learnable spectral operations.
• Generalization to External Data: The model has not been validated yet on external datasets acquired from other laboratories or clinics. This may affect generalizability across different staining protocols or microscope settings.

## Ethics statements

In this Manuscript no, human participants or animals their data or biological material, are not involved.

## CRediT authorship contribution statement

**Sannasi Chakravarthy SR:** Conceptualization, Data curation, Formal analysis, Investigation, Methodology, Writing – original draft, Writing – review & editing. **Harikumar Rajaguru:** Formal analysis, Project administration, Resources, Software, Supervision, Validation, Visualization, Writing – review & editing. **Rajesh Kumar Dhanaraj:** Funding acquisition, Formal analysis, Project administration, Resources, Software, Supervision, Validation, Visualization, Writing – review & editing. **Feslin Anish Mon:** Funding acquisition, Formal analysis, Project administration, Resources, Software, Supervision, Validation, Visualization, Writing – review & editing. **Dragan Pamucar:** Funding acquisition, Formal analysis, Project administration, Resources, Software, Supervision, Validation, Visualization, Writing – review & editing.

## Declaration of competing interest

The authors declare that they have no known competing financial interests or personal relationships that could have appeared to influence the work reported in this paper.

## Data Availability

Blood Cells Image Dataset
